# Supplementary material for: Evaluation of Multivariate Filters on Vibrational Spectroscopic Fingerprints for the PLS-DA and SIMCA Classification of Argan Oils from Four Moroccan Regions
Source: Molecules. 2023 Jul 27;28(15):5698. doi: 10.3390/molecules28155698 (PMC10419999; doi:10.3390/molecules28155698)
Supplement: Supplementary file 1 [file molecules-28-05698-s001.zip › molecules-2506258-supplementary.pdf]

Supplementary Materials

Table S1. The geographical origins, codes and number of samples used for the discrimination.

			Number of spectra in sub-sets	
**Geographical origin**	**Code**	**Number of samples**	**Training set**	**Test set**
**Agadir**	AG	27	54	27
**Essaouira**	ES	31	62	31
**Taroudant**	TA	24	48	24
**Tiznit**	TZ	11	22	11
**Total**		93	186	93

Table S2. Geographical parameters [46] and tocopherol contents [47] of the four provenances of Argan trees.

Provenance	Altitude (m)	Temperature min-max (°C)	Rainfall (mm/year)	Distance from the coast (km)	α-tocopherol (mg/kg)	β-tocopherol (mg/kg)	δ-tocopherol (mg/kg)
**Taroudant**	597	11.4–31.2	210	107	57.19	4.56	80.2
**Tiznit**	497	12.7–26.3	205	63	41.51	3.02	104.4
**Essaouira**	138	13.5–26.4	292	20	25.01	1.21	58.6
**Agadir**	103	12.9–24.5	264	22	34.98	1.61	71.0

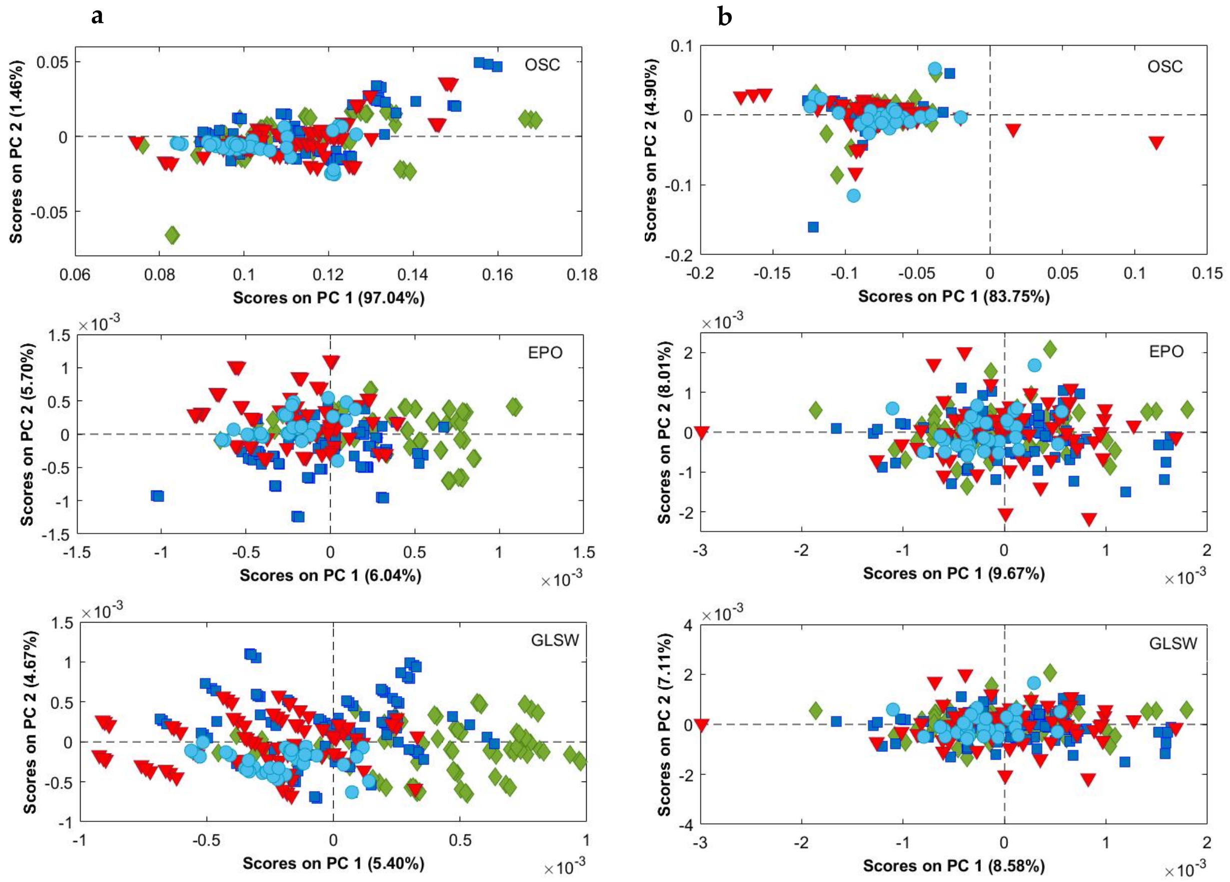

Figure S1. PCA score plots (PC1 vs PC2) of the MIR (a) and NIR (b) after random labelling and applying a filter method. Taroudant (▼); Essaouira (■); Agadir (◆); Tiznit (●).

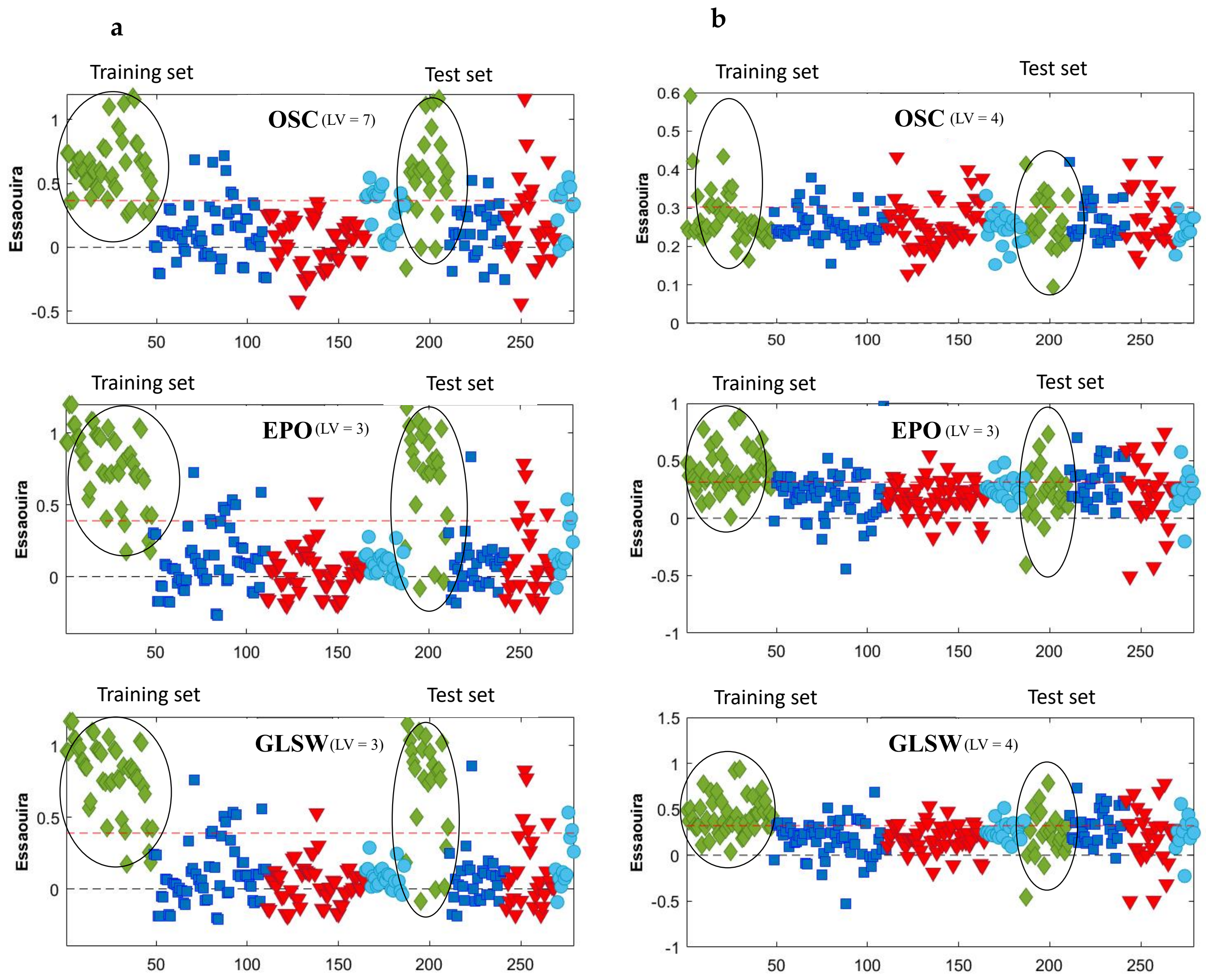

Figure S2. The prediction results for the Essaouira group from the randomly assigned classes data set applying a filter method and PLS-DA. (a) MIR and (b) NIR data. Agadir (■); Essaouira (◆); Taroudant (▼); Tiznit (●). The red dotted lines represent the classification threshold (or threshold value).
